# Hierarchical NiO/CMK-3 Photocathode for a *p*-Type Dye-Sensitized Solar Cell with Improved Photoelectrochemical Performance and Fast Hole Transfer

**DOI:** 10.3390/molecules25071638

**Published:** 2020-04-02

**Authors:** Jie Qu, Zhaoyang Fan, Hamed Mira, Jianan Wang, Amor M. Abdelkader, Shujiang Ding

**Affiliations:** 1 College of Chemistry and Chemical Engineering, Hunan Normal University, Changsha 410081, China; qujie@hunnu.edu.cn; 2 Xi′an Key Laboratory of Sustainable Energy Materials Chemistry, MOE Key Laboratory for Nonequilibrium Synthesis and Modulation of Condensed Matter, State Key Laboratory of Electrical Insulation and Power Equipment, Department of Applied Chemistry, School of Science, Xi’an Jiaotong University, Xi’an 710049, China; dingsj@xjtu.edu.cn; 3 Nuclear Materials Authority, Cairo 11381, Egypt; hamedmira@yahoo.com; 4 Department of Environmental Science and Engineering, State Key Laboratory of Multiphase Flow in Power Engineering, Xi’an Jiaotong University, Xi’an 710049, China; 5 Faculty of Science and Technology, Bournemouth University, Poole, Dorset BH12 5BB, UK

**Keywords:** NiO, CMK-3, Fast hole transport, Solar cell

## Abstract

The sluggish photoelectrochemical performance of *p*-type dye-sensitized solar cells (*p*-DSSCs) has hindered its commercial use. In this work, we introduce a novel hierarchical nanocomposite of NiO nanoparticles anchored on highly ordered mesoporous carbons CMK-3 (NiO/CMK-3). Using CMK-3 as a backbone effectively prevented the self-aggregation of NiO nanoparticles and subsequently increased the total specific surface area of the composite for more dye adsorption. The interconnected conductive networks of CMK-3 also served as a split-flow high-speed channel, which was beneficial for hole spin-flow to accelerate hole transfer. The hierarchical NiO/CMK-3 photocathode improved the photovoltaic conversion efficiency to 1.48% in a cell with a Cobalt(II)/(III) electrolyte and a PMI-6T-TPA dye.

## 1. Introduction

Conventional low-cost dye-sensitized solar cells based on *n*-type nanocrystalline TiO_2_ photoanodes (*n*-DSSCs) have attracted great interest for several years. The photocurrent is generated in these devices from dye-sensitized electron injection into *n*-type semiconductors, and conversion efficiency values as high as 12.3% have been reported [1]. Recently, dye-sensitized solar cells based on *p*-type semiconductors (*p*-DSSCs) have also attracted growing attention [2,3,4,5]. These have comparable theoretical efficiency to the *n*-DSSCs and similar operating principles with only the nature of the photoinjected charges being different. In addition, *p*-DSSCs can be combined with *n*-DSSCs to assemble a tandem solar cell (*pn*-DSSCs), with both electrodes becoming photoactivated. A theoretical upper limit efficiency of 43% can be achieved for such devices, which is much higher than that of single-junction solar cells [2,6]. However, the photoelectrical performance of *p*-DSSC is limited by intrinsically low voltages and currents. Such low photocurrents represent a major performance limitation in *p*-DSSCs. They are caused by (i) the fast charge recombination between the redox mediator dye and holes generated in the NiO and (ii) the overall low light-harvesting efficiency [7,8]. It is believed that the nature and morphology of nanocrystalline NiO has a great influence on the hole transfer processes that occur in the semiconductor electrode [9,10,11,12,13,14]. Although NiO nanoparticles were viewed as a promising material for *p*-DSSCs, they cannot fulfill all of the requirements for the target application. Two major limitations are the low intrinsic electric conductivity and hole diffusion coefficient of NiO films, which can hinder rapid charge flow and favor charge recombination between the excited state of the electrolyte and the excited electron within the dye molecule [15,16,17,18]. Besides, nanocrystalline NiO films have a relatively low Brunner–Emmett–Teller (BET) surface area and transparency, which restrains light harvesting and the penetration depth of light. Therefore, it is necessary to develop novel NiO nanostructures that can suppress the recombination and enhance the light absorption. Combining NiO nanoparticles with conductive nanomaterials, such as graphene, was a promising strategy to obtain high conductivity and larger surface area [19]. NiO hybrids with graphene, for example, yielded a significant improvement in the charge transfer, resulting in a reasonably high short-circuit photocurrent of 0.27 mA cm^–2^ and an open-circuit photovoltage of 105 mV [19]. However, the power conversion efficiency in this device is still very low. Highly crystalline nanostructured nickel (II) oxide microballs were developed with a high specific surface area and favorable optical properties. After sensitization with PMI-6T-TPA (perylenemonoimid-six thiophene-triphenylamine, the structure is shown in Figure S1), a record J_SC_ of 7.0 mA cm^–2^ and overall conversion efficiency of 0.43% were obtained [20]. There have been considerable efforts to improve the photoelectrochemical performance of *p*-DSSCs. Nevertheless, it is still not on a comparable level to that of *n*-DSSCs, which is due to the mismatching of dye, electrolyte, and *p*-type semiconductor.

**Figure 1:**
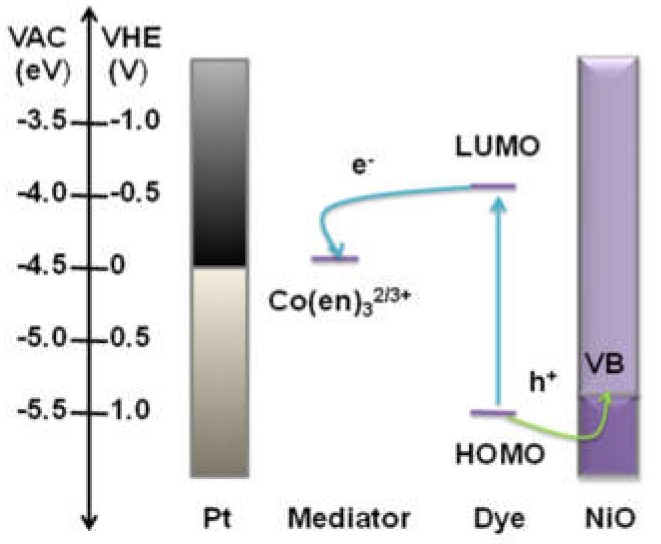
Energy level diagram for the prepared *p*-type dye-sensitized solar cell (*p*-DSSC).

In this article, a novel hierarchical NiO/CMK-3 nanocomposite was prepared using CMK-3 as a template. CMK-3 was utilized as a backbone to support NiO nanoparticles and was able to prevent self-aggregation and increase surface area effectively. The thin NiO layers with a large BET surface were found to be more favorable for dye adsorption, and the spaces constructed by NiO layers were able to accommodate more light. In addition, the inherent array channels of CMK-3 can be conducive for fast hole transport, and its interconnected conducting networks can be beneficial for hole spin-flow to accelerate hole transfer, serving as split-flow high-speed channels. A cobalt(II)/(III) electrolyte was used in our devices. This has a redox potential that is approximately 340 mV more negative than that of typical I^-^/I_3_^-^ electrolytes and should theoretically allow for increased open-circuit voltage. PMI-6T-TPA was used to sensitize the mesoporous NiO photocathode. An energy-level diagram for the as-prepared *p*-DSSC is shown in Figure 1. Together with this novel hierarchical NiO/CMK-3 nanocomposite, a photovoltaic conversion efficiency of 1.48% was achieved.

## 2. Results

**Figure 2:**
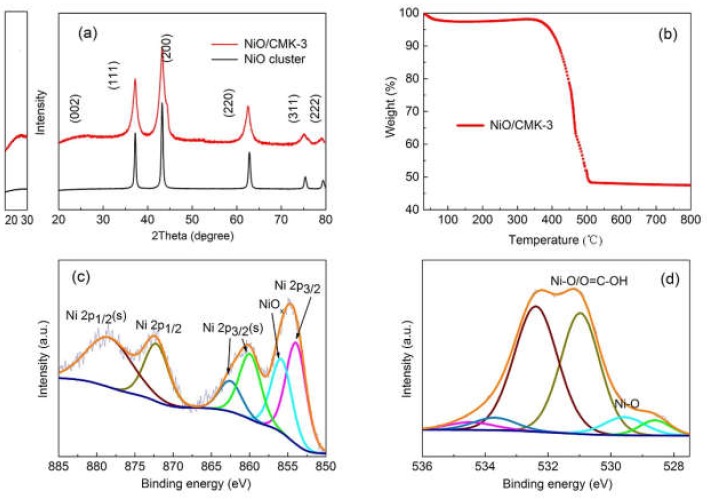
(**a**) XRD of the NiO/CMK-3 composite and NiO cluster; (**b**) Thermal gravimetric analysis (TGA) curve of NiO/CMK-3 composite; (**c**) Ni 2p XPS spectrum of NiO/CMK-3 composite, wine line: Ni^2+^ (Ni2p_1/2_ s), dark yellow line: Ni^2+^ (Ni2p_1/2_ ), dark cyan line: Ni^2+^ (Ni2p_3/2_ s), green line: Ni^2+^ (Ni2p_3/2_ s), cyan line: Ni^3+^, magenta line: Ni^2+^ (Ni2p_3/2_); (**d**) O1s XPS spectrum of NiO/CMK-3 composite, wine line: O^2+^ (NiO_x_), dark yellow line: O^2+^ (NiO, C=O), dark cyan line: O^2+^ (H_2_O), green line: O^2+^ (NiO), cyan line: O^2+^ (NiO).

X-ray diffraction (XRD) patterns of the NiO and NiO/CMK-3 composite samples are shown in Figure 2a. All the diffraction peaks match well with cubic rock salt NiO (JCPDS File No. 78-0643). The sharp peaks indicate high levels of crystallinity of NiO. A characteristic diffraction peak of graphitic carbon appears at around 25°, which can be attributed to CMK-3 [21]. The broad peak indicates that CMK-3 does include a stacked crystalline graphitic phase as a component. The conductivity of CMK-3 with such a graphitic phase can be improved, and CMK-3 can serve as a shuttle to enhance electron transport and suppress recombination [19]. The thermal gravimetric analysis (TGA) curve (Figure 2b) shows the thermal stability of the NiO/CMK-3 composite. The slight weight loss at temperatures under 100 °C can be attributed to the evaporation of residual water from the preparation process. A more substantial weight loss step occurs between 350–510 °C and can be attributed to the combustion of crystallized CMK-3 [22]. The content of NiO in this composite is estimated from the TGA curve to be 51.2 wt%.

X-ray photoelectron spectroscopy (XPS) was used to analyze the chemical compositions and the possible bonds between the constituents of the prepared NiO/CMK-3 nanocomposite. All elements (Ni, O, and C) relevant to NiO/CMK-3 nanocomposite were observed (Figure S3a). Figure 2c,d show the high-resolution spectra of Ni2p and O1s. There are two regions for Ni2p spectra; one is the Ni2p_3/2_ of 851–866 eV, the other is Ni2p_1/2_ spin-orbit levels of 870–885 eV. The main peak in the Ni2p_3/2_ region at 854 eV is indexed to the Ni^2+^ in the Ni-O bonding configuration [23,24,25,26]. While the shoulder peak at 856 eV stems from the Ni^2+^-vacancy induced Ni^3+^ ion or excess oxygen from compounds such as oxyhydroxides (NiIIIOOH) [27,28]. To further investigate the species, as described in the experimental section, the nanocomposite samples were calcined at 600 °C. Under this thermal treatment, NiOOH species can be obviously reduced. So Ni^3+^ is mainly induced by Ni^2+^-vacancy, which can generate *p*-type conductivity in the NiO_x_ [29]. As shown in Figure 2d, the peaks at 528.6 and 529.6 eV are due to lattice oxygen of NiO. The O1s peak at 531 eV is attributed to O^2^^–^ of NiO and C=O, while the peak at 532.5 eV is usually associated with defects of NiO [26,30,31]. Figure S3b displays the C 1s spectra. The peaks at 283.8 and 284.2 eV correspond to C–C or C=C. While the other three peaks at 285.4, 286.9, and 287.9 are attributed to C=O/C-O, COO, and C=O, respectively [32].

**Figure 3:**
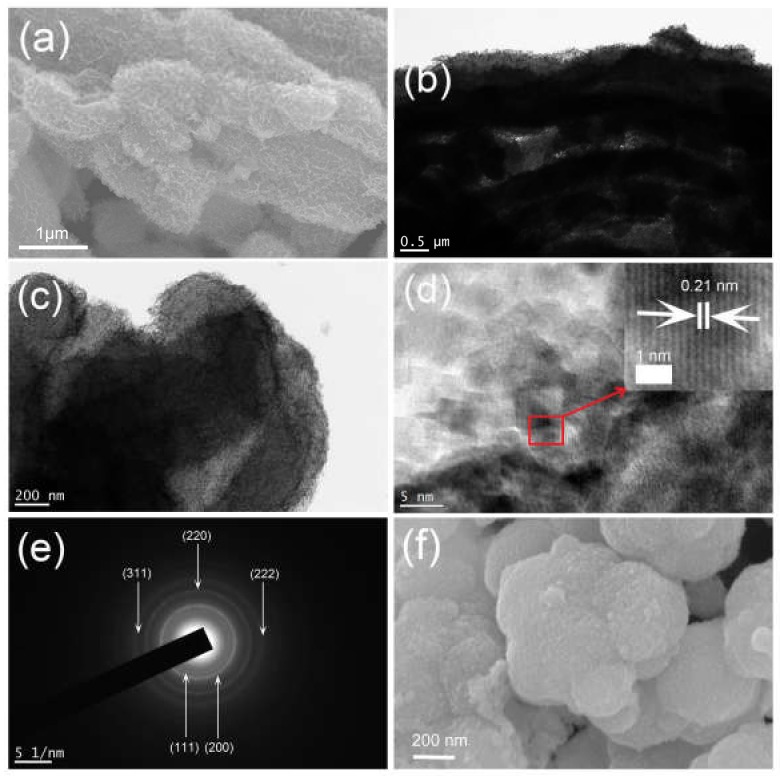
SEM (**a**), TEM (**b**,**c**), HRTEM (**d**), and SAED images (**e**) of NiO/CMK-3 composite; SEM image (**f**) of NiO cluster.

**Figure 4:**
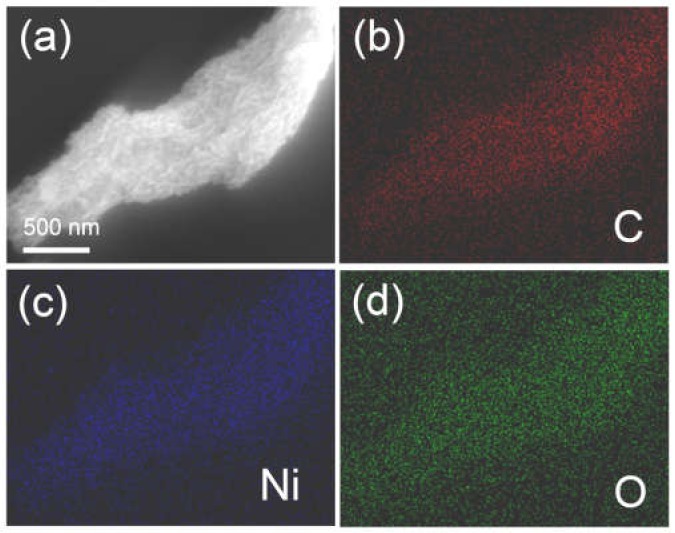
SEM image (**a**) of NiO/CMK-3 composite and the corresponding elemental mapping images of carbon (**b**), nickel (**c**), and oxygen (**d**).

The structure of the CMK-3 and the schematic illustration of the synthetic process of the Ni-precursor/CMK-3 are shown in Figure S2. The morphology of the as-prepared NiO/CMK-3 nanocomposite was determined from scanning electron microscopy (SEM) and transmission electron microscopy (TEM) images (Figure 3). Figure 3a indicates a uniform distribution of NiO on CMK-3. The TEM images indicate that the NiO layer is wrapped around a CMK-3 backbone. The NiO layer is composed of nanoparticles with diameters in the range of 3–5 nm (Figure 3d). The NiO aggregates together to form a solid cluster that combine to form a continuous layer (Figure 3f). The interplanar distance measured from a high-resolution transmission electron microscopy (HRTEM) image is 0.21 nm, corresponding to the (200) plane of cubic NiO [33]. The selected-area electron diffraction (SAED) pattern is shown in Figure 3e. Five diffraction rings are presented in the picture, which can be attributed to the (111), (200), (220), (311), and (222) planes of the NiO lattice, respectively. In addition, the morphology of the NiO cluster was also tested. The elemental mapping (Figure 4) further confirmed the uniform distribution of the NiO nanoparticles in the composite. C is located at the center of the NiO/CMK-3 composite, forming the core, while Ni and O elements hold a wider area, which verifies that NiO covers the surface of CMK-3.

## 3. Discussion

**Figure 5:**
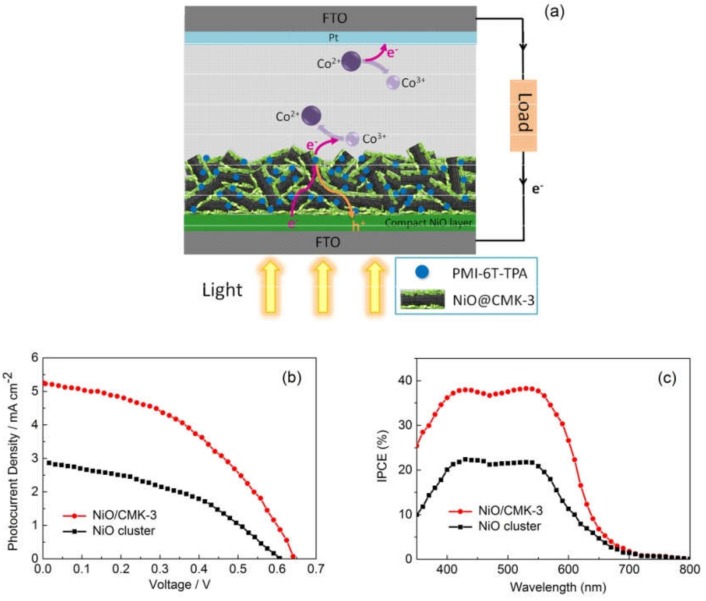
(**a**) *p*-DSSC structure and scheme for the electron-transfer processes occurring in the *p*-DSSC; (**b**) Current–density–voltage characteristics of *p*-DSSCs; (**c**) IPCE spectra of *p*-DSSCs sensitized by PMI-6T-TPA.

To investigate the photoelectrochemical performance of the NiO/CMK-3 composite, we assembled a *p*-DSSC device in which NiO was used to fabricate the photocathode. The *p*-DSSC structure and the electron-transfer processes are shown in Figure 5a. It should be noted that double-layered NiO was used in this device. A compact NiO blocking layer was first deposited on fluorine-doped tin oxide (FTO) glass, and then a hierarchical NiO/CMK-3 or NiO-clusters layer was applied. A compact NiO blocking layer can effectively improve the overall photoelectrical performance (for example, short circuit current density: J_sc_, open circuit voltage: V_oc_, fill factor: FF and photovoltaic conversion efficiency: η), and suppress hole transfer recombination, so as to increase hole collection efficiency [2,11,34].

Figure 5b shows the photocurrent–voltage curves of *p*-DSSCs based on-hierarchical NiO/CMK-3 tested under simulated sun illumination (AM 1.5). The photocurrent–voltage curve of a controlled device fabricated using NiO-cluster photocathodes is also illustrated in Figure 5b for comparison. The device based on hierarchical NiO/CMK-3 shows a higher J_sc_ (5.25 mA cm^–2^) than that based on NiO clusters (2.86 mA cm^–2^). The higher J_sc_ of the NiO/CMK-3 can be explained by the high specific surface area of the nanocomposite (508 m^2^ g^–^^1^), which is far higher than that previously reported [9,19,20,35,36,37]. CMK-3 has a high specific surface of 1091 m^2^ g**^–^**^1^, and in the hierarchical structure acts as a template that effectively prevents the self-aggregation of NiO. NiO clusters, on the other hand, are easily agglomerated, with a total specific surface area of only 45 m^2^ g^–^^1^. With a higher available surface area, far more PMI-6T-TPA molecules can attach onto the hierarchical NiO/CMK-3 and improve the short circuit current density of the solar cell. The V_oc_ values are determined by the difference of the quasi-Fermi level of NiO and redox potential of the electrolyte. In the current work [Co(en)_3_]^2+/3+^ is used as a redox mediator, which shows a much higher vacuum energy value with respect to NiO and should result in higher V_oc_ [38]. The V_oc_ values of both samples here are above 600 mV, with the value of the hierarchical NiO/CMK-3 device being 34 mV higher than for that based on NiO clusters. As reported previously, hole transport processes can also influence the V_oc_ in *p*-DSSCs. When hierarchical NiO/CMK-3 was used as a photocathode, injected holes are transferred rapidly from NiO to CMK-3, acting as a split-flow high-speed channel for faster hole transport through the external circuit. This allows for favorable energy level alignment and strong interactions between the NiO and the CMK-3 backbone. During this process, hole recombination is suppressed and results in an increased value of V_oc_ and enhances the overall device performance. The details of J_sc_, V_oc_, FF, and η are listed in Table 1. Consequently, a higher photovoltaic conversion efficiency of 1.48% is obtained for the device based on the hierarchical NiO/CMK-3 nanocomposite, which is double that of the device based on NiO clusters (0.71%). 

*p*-DSSC based on NiO/CMK-3 shows a two-fold increase in J_sc_ than that based on NiO cluster. Such trends were also reflected in the incident photon-to-charge carrier conversion efficiency (IPCE) spectra, as shown in Figure 5c. A broad plateau between 400 and 570 nm was observed for both samples and trailed off up to 700 nm [20]. IPCE values for NiO/CMK-3 based *p*-DSSCs are generally higher than those of NiO cluster-based *p*-DSSCs. A further increase in dye loading may result in the improvement of the IPCE value. The highest value close to 40% occurred for NiO/CMK-3 based *p*-DSSCs. Finally, the real tested J_sc_ values are very close to the theoretically calculated J_sc_ values. 

**Figure 6:**
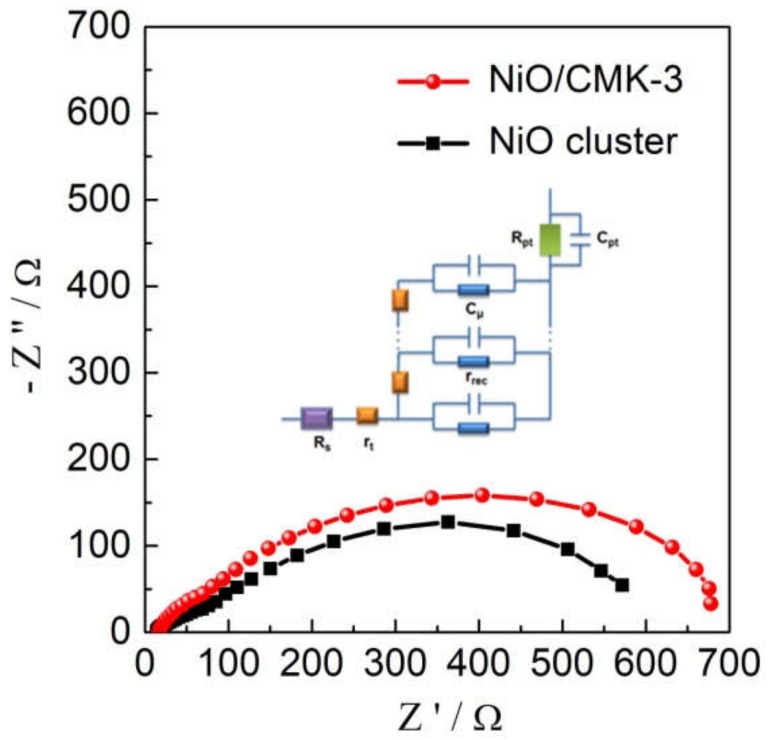
Nyquist plots of the *p*-DSSCs made of two nanomaterials and the insert is the equivalent circuit for the impedance spectrum. R_s_: serial resistance; R_t_: transport resistance of the film; R_rec_: the recombination resistance of the film; R_pt_: charge-transfer resistance of Pt electrode; C_µ_: distributed capacitance of *p*-semiconductor; C_pt_: the double-layer capacitance of the platinized counter electrode.

**Figure 7:**
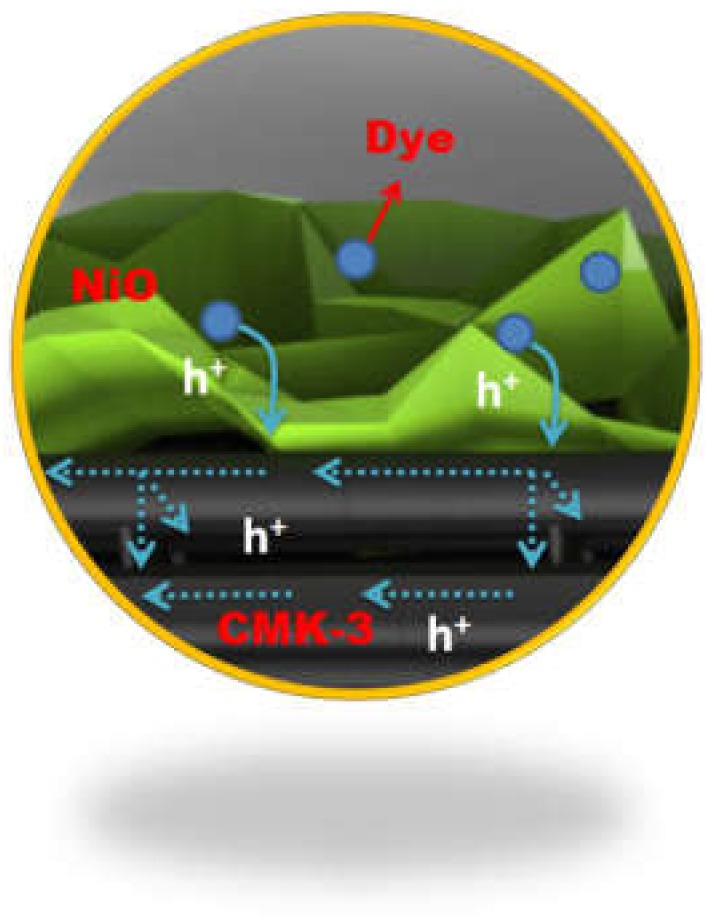
The schematic representation of the hole transfer path in the NiO/CMK-3 structure.

Electrochemical impedance spectroscopy (EIS) was carried out under the same conditions as the photocurrent–voltage (I–V) testing to further understand the charge transfer processes in the NiO/CMK-3 nanocomposite. The Nyquist plots (Figure 6) show two distinct semicircles; one in the low-frequency range and one in the high-frequency range. The low-frequency semicircle on the right of the plot corresponds to hole transport in the mesoscopic NiO film (R_t_) and the back reaction occurring at the NiO/dye/electrolyte interface (R_rec_). The second semicircle in the high-frequency range, on the left of the plot, can be assigned to the electrolyte/Pt electrode interface (R_pt_) [2,14]. The above detailed fitting parameters are achieved by a transmission line equivalent circuit as summarized in Table 2, Figure S5 and Table S1 [2,14,39]. R_pt_ values of the two *p*-DSSCs are relatively similar, which might be due to the comparable device structure. The R_t_ values obtained, however, are distinctly different, the value determined for the NiO/CMK-3 device is significantly lower than that for the NiO-cluster device. This indicates much faster hole transport in the NiO/CMK-3 film, which may occur due to the split-flow high-speed route of the CMK-3 backbone. The R_rec_ value of NiO/CMK-3 is much higher than that of the NiO cluster, implying prohibited recombination at the NiO/CMK-3/dye/electrolyte interface. The hole lifetime (τ_h_) can be determined using the Equation $\tau_{h}={1/2\pi f}_{\min}$ from the IMVS results, while hole transport time (τ_th_) can be determined using the Equation $\tau_{th}={1/2\pi f}_{\min}$ from the IMPS results (*f*_min_ corresponds to the frequency at the bottom of the spectrum plot) [40,41]. The IMPS and IMVS results are shown in Figure S4, and the calculated τ_h_ and τ_th_ values are shown in Table 2. The charge collection efficiency (*η_cc_*) can be calculated from the hole transport time (τ_th_) and lifetime (τ_h_) using the Equation: $\eta_{cc}=1-\tau_{th}/\tau_{h}$ [14]. The calculated parameters are summarized in Table 2. The hole lifetime of NiO/CMK-3 is longer than that of the NiO cluster, indicating a lower rate of recombination, which is beneficial for *p*-DSSCs. The shorter hole transport time for NiO/CMK-3 than that of the NiO cluster can be attributed to the high electrical conductivity and the well-designed structure of CMK-3. The τ_h_/τ_th_ ratio for NiO/CMK-3 is 7.1, more than double that for the NiO-cluster device and is an indication of reduced recombination. As schematically demonstrated in Figure 7, the inside channels of CMK-3 provide a direct hole transport route, just like the high speed route, which can shorten the hole transport time. Moreover, the interconnected nanorods of CMK-3 are more beneficial for fast hole transfer. The holes will travel into other nanorods when there are many more holes. Fast hole transport can also reduce the recombination with the electrolytes. In short, the favorable morphological characteristics lead to enhancement of the photoelectrochemical performance. The hierarchical NiO/CMK-3 sample exhibits an enhanced charge collection efficiency of 85%, which can be attributed to reduced recombination and faster hole transport. The hole diffusion length (*L_n_*), a characteristic length scale, is defined by Equation ($L_{n}=d\sqrt{\tau_{h}/\tau_{th}}$). It is obvious that NiO/CMK-3 shows longer *L_n_* compared to the NiO cluster. It is well-known that for an efficient hole transport *L_n_* should be larger than the film thickness; the *L_n_* value of NiO/CMK-3 is more than two times larger than the 2 µm thickness of the NiO film. The above results are in good agreement with those previously reported [34], which also indicates that hole transport and recombination kinetics are to some extent related to the intrinsic properties of the photocathode semiconductors.

## 4. Materials and Methods 

All chemicals used in this article were bought from Xi’an Kequan Laboratory Equipment Co.,Ltd (Xi’an, Shanxi province, China), except for the PMI-6T-TPA, which was bought from Suzhou Lvguang Chemical Co.,Ltd (Suzhou, Jiangsu province, China). 

### 4.1. Preparation of Hierarchical NiO/CMK-3 Nanocomposite and NiO Clusters 

Mesoporous CMK-3 was prepared by the procedure previously reported by Jun et al. [42]. In a typical synthesis of NiO/CMK-3 composite, 15 mg of CMK-3 were dispersed into 40 mL of deionized water and sonicated for 5 min. This was followed by the addition of 0.25 mmol of Ni(NO_3_)_2_·6H_2_O, 0.25 mmol of hexamethylenetetramine (HMT), and 0.025 mmol of citric acid trisodium salt dehydrate, in order to form a homogeneous green solution. This solution was then sonicated for a further 5 min and transferred into a 100 mL round-bottom flask. After magnetically stirring for 6 h at 90 °C in an oil bath, the solution was then left to cool down to room temperature. A black precipitate was then obtained by centrifugation, which was washed thoroughly with ethanol and dried at ambient temperature overnight. The dried product was then further thermally treated at 600 °C under N_2_ atmosphere for 3 h, at a heating rate of 1 °C min^–1^.

NiO clusters were prepared via a similar procedure, without the addition of CMK-3.

### 4.2. Materials Characterization

The structure of the NiO nanomaterials were obtained using a Rigaku X-ray diffractometer (D/max-2500) with CuKa radiation (λ = 0.15418 nm) at 40 mA and 40 kV. The data were collected in the 2θ range of 5–80^°^ with a scanning rate of 5^°^ min^–1^ and a step size of 0.05^°^. The morphology was observed on a field-emission scanning electron microscope (FESEM; HITACHI, su-8010, Tokyo, Japan) and a transmission electron microscope (TEM; JEOL, JEM-2100, Tokyo, Japan). The elemental distribution was investigated on a scanning transmission electron microscope (STEM; JEOL, ARM200F, Japan), with an energy dispersive X-ray (EDX) spectrometer. X-ray photoelectron spectroscopy (XPS) analyses were tested with a PHI5300 analyzer (PerkinElmer, Waltham, MA, USA) with AlK radiation. Thermal Gravimetric Analysis (TGA, SDT Q600, New Castle, DE, USA) was carried out in an air atmosphere at a rate of 10 ^°^C min^–1^. N_2_ adsorption-desorption data were measured using a NOVA 2000e (Quantachrome, Boynton Beach, FL, USA) instrument, and the specific surface area was evaluated using the Brunauer–Emmett–Teller (BET) method. 

### 4.3. Preparation of the NiO and NiO/CMK-3 Films

The photocathode semiconductor films were made by a two-step method on fluorine-doped tin oxide (FTO) conductive glass. NiO nanoparticles were first coated onto FTO using magnetron sputtering to form the compact layer. This was followed by applying a layer of either NiO/CMK-3 nanocomposite or NiO cluster, acting as the second light-scattering layer. 

### 4.4. Fabrication and Characterization of the p-DSSC Devices

The prepared photocathodes were immersed in a PMI-6T-TPA dye solution (0.2 mM in DMF) for 12 h. The photocathodes were then sandwiched together with platinized counter electrodes and sealed with Surlyn film. The electrolyte used was composed of 0.07 M [Co(en)_3_](BF_4_)_3_, 0.3 M [Co(en)_3_](BF_4_)_2_, and 0.1 M lithium bis (trifluoromethanesulfonylimide) (LiTFSI) in acetonitrile. The active electrode area employed was approximately 0.16 cm^2^. A Zahner CIMPS-2 electrochemical workstation (Kronach, Germany) together with a Trusttech CHF-XM-500W source (Beijing, China) under simulated Sun illumination (Global AM 1.5, 100 mW cm^–2^) were used to produce photocurrent–voltage (I–V) curves and electrochemical impedance spectra (EIS) (100 kHz to 0.1 Hz, 10 mV perturbation). Intensity-modulated photovoltage spectroscopy (IMVS) and intensity-modulated photocurrent spectroscopy (IMPS) were carried out using a Zahner CIMPS-2 system. The lamp assembly was fitted with a blue light-emitting diode (LED) (470 nm) and driven by a PP210 (Zahner) frequency response analyzer, with a frequency range from 1000 Hz to 0.01 Hz. The incident photon-to-electron conversion efficiency (IPCE) experiments were performed using a system consisting of a Xe lamp (300 W) with a monochromator (Oriel 74100, Newport Corp., Irvine, CA, USA).

## 5. Conclusions

In summary, a hierarchical NiO/CMK-3 nanocomposite was synthesized using CMK-3 as a templet and backbone to support and avoid the NiO nanoparticles aggregation. The hierarchical nanostructure possesses a large surface area that can absorb many more dye molecules and trap more light. A *p*-DSSC device fabricated using NiO/CMK-3 photocathodes showed photovoltaic conversion efficiency as high as 1.48%. The device also showed superior hole transport kinetics, fast hole transport, long hole lifetime, prohibition of recombination, and hole collection efficiency. The CMK-3 with so many channels can act as a split-flow high-speed channel for fast hole transport through the external circuit. The novel hierarchical NiO/CMK-3 nanocomposite is expected to be potentially applied as a photocathode for *p*-DSSCs. 

## Figures and Tables

**Table 1:** Detailed photovoltaic parameters of the as-prepared *p*-DSSCs.

Sample	*J*_sc_ (mA cm^–2^)	*V*_oc_ (mV)	*FF*	*η* (℅)
NiO/CMK-3	5.25	641	0.44	1.48
NiO cluster	2.86	607	0.41	0.71

**Table 2:** Fitted and calculated data from EIS, IMPS, and IMVS spectra of *p*-DSSCs based on NiO/CMK-3 and NiO-cluster photocathodes.

Sample	R_pt_ (Ω)	R_t_ (Ω)	R_rec_ (Ω)	*τ_th_* (ms)	*τ_h_* (ms)	*η_cc_*	*L_n_* (µm)
NiO/CMK-3	37	89	614	11	78	0.86	5.33
NiO cluster	41	197	508	20	52	0.62	3.22

## Supplementary Materials

The following are available online at https://www.mdpi.com/1420-3049/25/7/1638/s1, Figure S1: The structure of the PMI-6T-TPA sensitizer, Figure S2: Schematic illustration of the synthetic process of the Ni-Precursor/CMK-3, Figure S3: (a) XPS survey scan of NiO/CMK-3 composite; (b) C1s XPS spectrum of NiO/CMK-3 composite, Figure S4: Short-circuit IMPS response (a), (c) and IMVS response (b), (d) for NiO/CMK-3 and NiO cluster, respectively, Figure S5: Fitted EIS plot of p-DSSCs made of two nanomaterials and the insert is calculated parameters, Table S1: The fitting errors of corresponding EIS parameters.
