# Supplementary material for: Hierarchical NiO/CMK-3 Photocathode for a p-Type Dye-Sensitized Solar Cell with Improved Photoelectrochemical Performance and Fast Hole Transfer
Source: Molecules. 2020 Apr 2;25(7):1638. doi: 10.3390/molecules25071638 (PMC7181050; doi:10.3390/molecules25071638)
Supplement: Supplementary file 1 [file molecules-25-01638-s001.pdf]

Supporting Information

Hierarchical NiO/CMK-3 Photocathode for a *p*-Type Dye-Sensitized Solar Cell with Improved Photoelectrochemical Performance and Fast Hole Transfer

Jie Qu [1], Zhaoyang Fan [2,*], Hamed Mira [3], Jianan Wang [2,4,*], Amor M. Abdelkader [5,*] and Shujiang Ding [2]

[1] College of Chemistry and Chemical Engineering, Hunan Normal University, Changsha 410081, China; qujie@hunnu.edu.cn
[2] Xi'an Key Laboratory of Sustainable Energy Materials Chemistry, MOE Key Laboratory for Nonequilibrium Synthesis and Modulation of Condensed Matter, State Key Laboratory of Electrical Insulation and Power Equipment, Department of Applied Chemistry, School of Science, Xi'an Jiaotong University, Xi'an 710049, China; dingsj@xjtu.edu.cn
[3] Nuclear Materials Authority, Cairo 11381, Egypt; hamedmira@yahoo.com
[4] Department of Environmental Science and Engineering, State Key Laboratory of Multiphase Flow in Power Engineering, Xi'an Jiaotong University, Xi'an 710049, China
[5] Faculty of Science and Technology, Bournemouth University, Poole, Dorset BH12 5BB, UK
* Correspondence: z.y.fan@stu.xjtu.edu.cn (Z.F.); wangjn116@xjtu.edu.cn (J.W.); aabdelkader@bournemouth.ac.uk (A.M.A.)

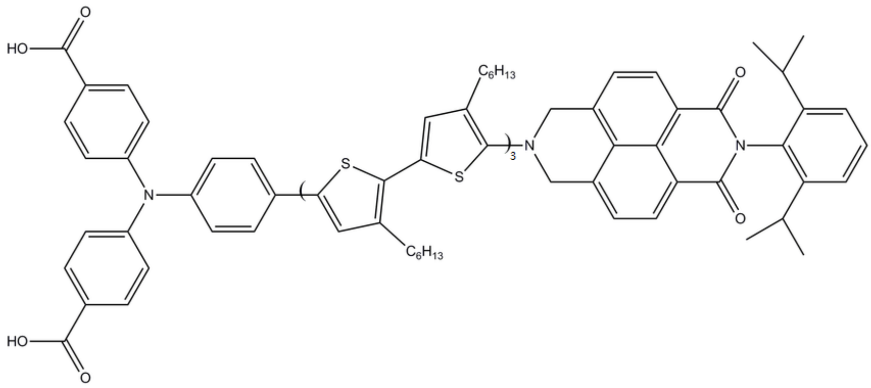

Figure S1. The structure of the PMI-6T-TPA sensitizer.

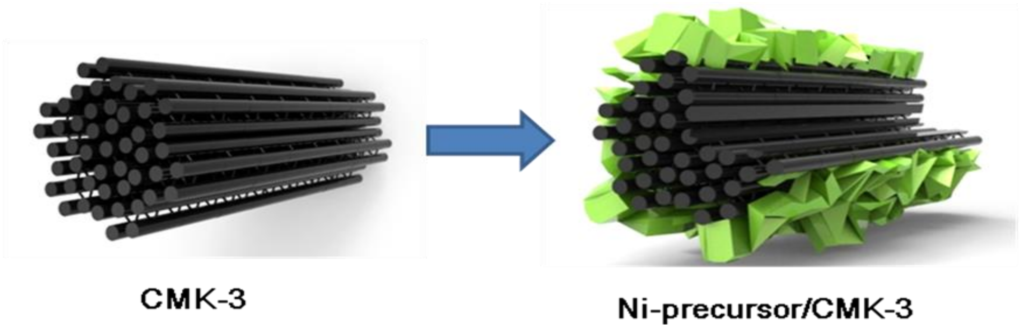

Figure S2. Schematic illustration of the synthetic process of the Ni-Precursor/CMK-3.

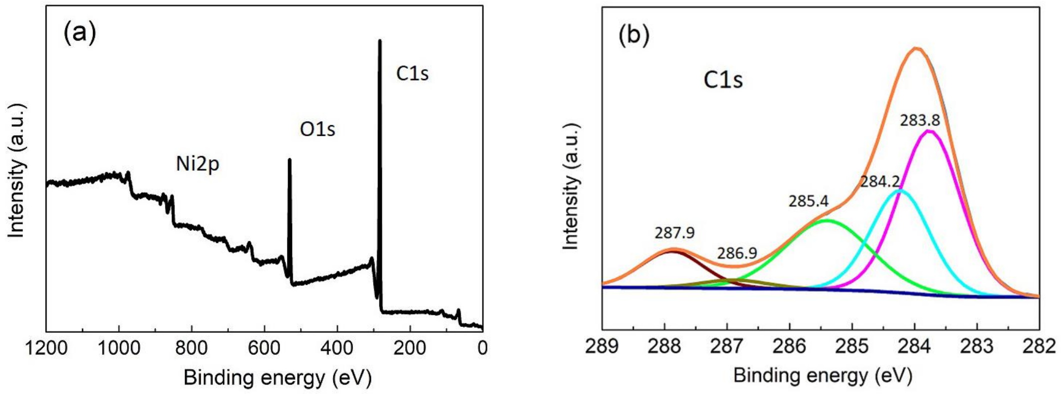

Figure S3. (a) XPS survey scan of NiO/CMK-3 composite; (b) C1s XPS spectrum of NiO/CMK-3 composite.

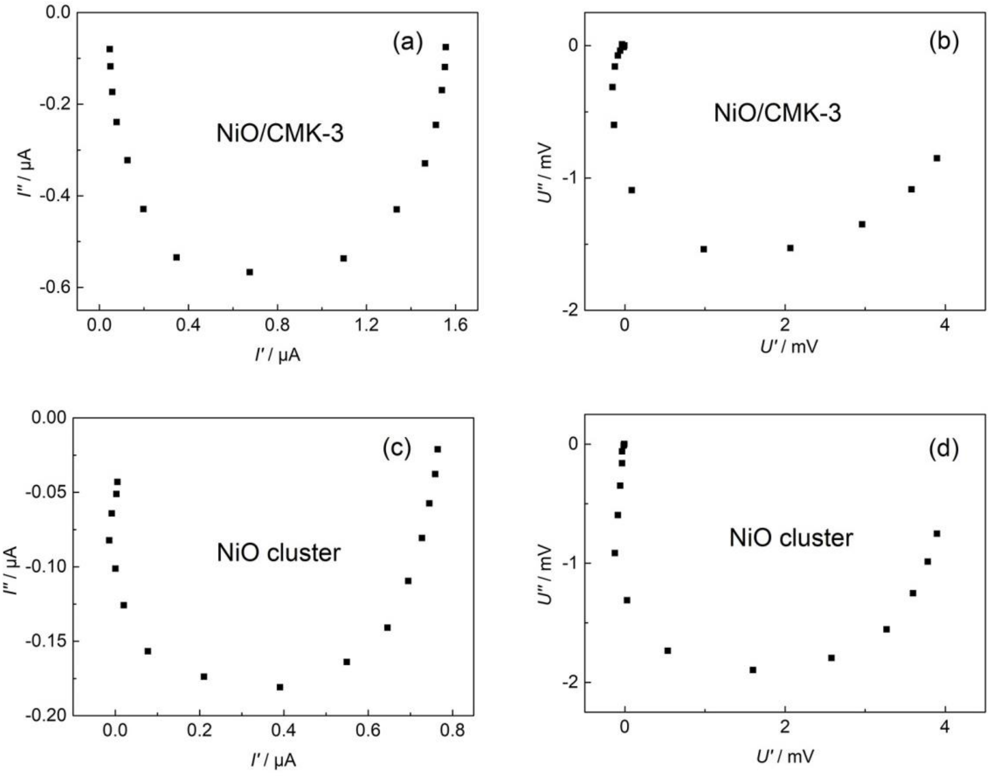

Figure S4. Short-circuit IMPS response (a), (c) and IMVS response (b), (d) for NiO/CMK-3 and NiO cluster, respectively.

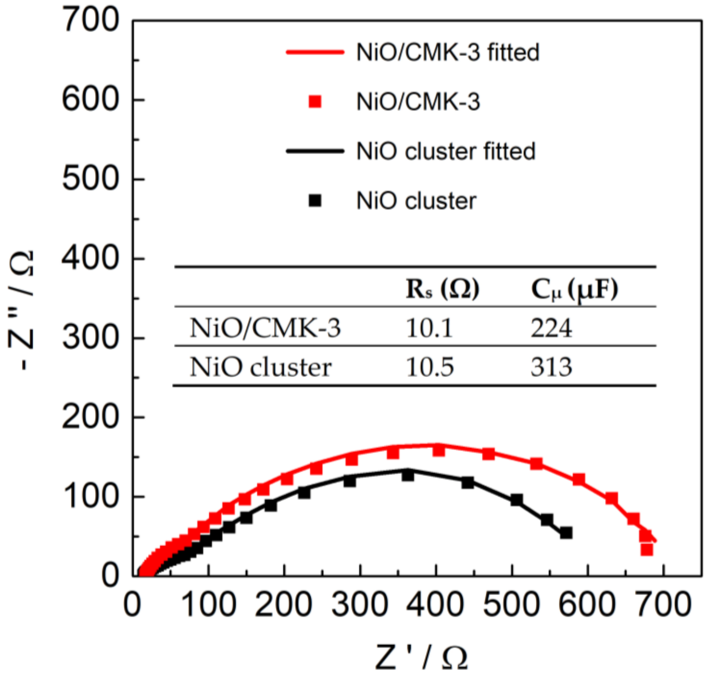

Figure S5. Fitted EIS plot of p-DSSCs made of two nanomaterials and the insert is calculated parameters.

Table S1. The fitting errors of corresponding EIS parameters.

Sample	R_{pt} (%)	R_t (%)	R_{rec} (%)	R_s (%)	C_μ(%)
NiO/CMK-3	0.17	3.94	5.02	0.21	4.79
NiO cluster	1.46	0.73	2.86	0.47	5.24
